# Assessing Raman Spectroscopy as a Prescreening Tool for the Selection of Archaeological Bone for Stable Isotopic Analysis

**DOI:** 10.1371/journal.pone.0098462

**Published:** 2014-07-25

**Authors:** Siân E. Halcrow, Jeremy Rooney, Nancy Beavan, Keith C. Gordon, Nancy Tayles, Andrew Gray

**Affiliations:** 1 Department of Anatomy, University of Otago, Dunedin, New Zealand; 2 Department of Chemistry, University of Otago, Dunedin, New Zealand; 3 Department of Preventative and Social Medicine, University of Otago, Dunedin, New Zealand; The Pennsylvania State University, United States of America

## Abstract

Stable isotope analyses for paleodiet investigations require good preservation of bone protein, the collagen, to obtain reliable stable isotope values. Burial environments cause diagenetic alterations to collagen, especially in the leaching of the organic bone content. The survival of bone protein may be assessed by the weight % collagen, % carbon and % nitrogen yields, but these values are achieved only after destructive chemical processing. A non-destructive method of determining whether bone is suitably preserved would be desirable, as it would be less costly than chemical processing, and would also preserve skeletal collections. Raman analysis is one such potential non-destructive screening method. In previous applications, Raman spectroscopy has been used to test both the alteration of the mineral portion of bone, as well as to indicate the relative amount of organic material within the bone structure. However, there has been no research to test the relationship between the Raman spectroscopic results and the survival of bone protein. We use a set of 41 bone samples from the prehistoric archaeological site of Ban Non Wat, Northeast Thailand, to assess if Raman spectroscopy analysis of the organic-phosphate ratio has a significant correlation with the weight % collagen, and carbon and nitrogen yields obtained by isotopic analysis. The correlation coefficients are highly statistically significant in all cases (r = 0.716 for collagen, r = 0.630 for carbon and r = 0.706 for nitrogen, p≤0.001 for all) with approximately or close to half of the variation in each explained by variation in the organic-phosphate ratio (51.2% for collagen, 39.6% for carbon, and 49.8% for nitrogen). Although the Raman screening method cannot directly quantify the extent of collagen survival, it could be of use in the selection of bone most likely to have viable protein required for reliable results from stable isotope analysis.

## Introduction

Stable isotope analysis of bone protein (collagen) and bone mineral (apatite) have been used extensively to determine the diet and movement of past people [1]–[6]. Experimental data have indicated that different bone tissues reflect different components of the diet [7], [8]. Stable isotope values in bone collagen are mainly influenced by the protein portion of the diet, while the bone and tooth enamel mineral component (carbonated-hydroxyapatite or apatite), is reflective of a mixture of dietary protein, carbohydrates and fats. Quantitative estimates of dietary influences from various sources (e.g. terrestrial or marine, and plant or animal protein components) can also be made from analysis of bone collagen and apatite. Bone collagen and bone apatite are remodelled during life and thus their isotopic composition reflects dietary averages over a certain time period [6], [9], [10].

The isotopic signatures of bone collagen and apatite can be compromised by poor preservation. Among the types of burial environments that most severely alter bone protein and bone apatite are conditions found in the monsoonal tropics, where heat and alternating wet and dry seasons leach bone protein and recrystallise bone apatite. The preservation state of collagen is particularly important in the application of stable isotope analysis for dietary studies. The isotopic values of carbon and nitrogen in bone protein are derived from the metabolism of food sources, but diagenetic changes to bone protein from the burial environment can alter the original diet-derived isotopic signature [2], [11]. These diagenetic changes include the breakdown of the hydrogen bonds in bone collagen from excessively hot temperatures and extremes of aridity or moisture [12] or the intrusion of exogenous carbon from humic and fulvic acids (fulvic acids are humic acids of lower molecular weight and higher oxygen content) in burial soils. As collagen deteriorates there is a reduction in % nitrogen, in part due to the denaturing of the collagen helix and cleaving-off of amino acids, which are more susceptible to degradation [13]. As each amino acid which constitutes the whole collagen protein has a δ^13^C and δ^15^N signature arising from its source and metabolic generation [14], [15], the loss of certain amino acids during protein degradation may alter the overall stable isotope value of the collagen of interest for dietary analysis [10], [16]–[18] with a possible shift of δ^13^C to depleted values and an enrichment of δ^15^N signals [13]. The isotopic abundance of a sample is preceded by the notational symbol δ, which is called the delta ratio. The delta ratio of a sample is calculated by the formula:


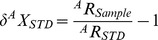
 [19]. Where δ expresses the abundance of isotope A of element X in a sample relative to the abundance of that same isotope in an isotopic standard, which is analysed along with the unknown samples. Because of this potential collagen degradation, paleodietary studies firstly assess collagen integrity by one or more screening methods. A suite of indicators can be used to assess collagen survival and presence of exogenous carbon, including techniques such as % collagen yield (weight % collagen derived from undemineralised bone), collagen amino acid composition, weight % carbon and nitrogen [2], [6], [11], [20]–[22], and carbon to nitrogen (CN) ratios, indicative of preserved bone protein in the 2.9–3.6 range [23]. Each of these screening methods requires bone to be destroyed during chemical preparation to isolate the collagen. It would be useful if a screening method was available to select the best preserved bone for stable isotope analyses from often precious collections.

Archaeological research in tropical areas, including Asia, have long faced a limited availability of sound skeletal material for isotopic analysis. Recent research by King at al. [24] on the bone of 40 adults from the prehistoric archaeological site of Ban Non Wat in Northeast Thailand used Raman spectroscopy to qualitatively assess the extent of diagenesis. The research investigated chemical changes in bone such as fluoridation, carbonate substitution in the apatite, and the presence or absence of collagen [24]. In King et al.'s [24] study, Raman results were interpreted as indicating a total leaching of bone protein from all samples considered. However, % collagen, and % carbon or % nitrogen yield derived from stable isotope analysis were not produced to compare with the qualitative measures of Raman spectroscopy. The aim of the present paper is to analyse how Raman organic-phosphate ratios correlate with actual percentages of collagen, carbon and nitrogen retrieved from bone samples to assess the usefulness of Raman spectroscopy as a tool for detecting the protein component of bone. There was a statistically significant correlation between the Raman organic-phosphate ratio and % collagen, % carbon and % nitrogen, which indicates that Raman spectroscopy could be used in the future as a pre-screening tool for preservation of the organic component of bone for stable isotope analyses.

### Raman spectroscopy

Raman spectroscopy involves a sample being irradiated with a laser, with some of the resulting scattered photons containing information on the vibrational energy levels of the sample. These data may be used to quantify the constituents of a sample [25].

Uses of Raman spectroscopy include the examination of bone quality and health in patients by assessing organic-phosphate ratios [26], lamellar bone orientation and bone composition [27], and mechanical stress on collagen fibrils in bone [28]. Raman spectroscopy can also be used in forensic contexts to determine the post-mortem interval of death by monitoring organic material loss in bones [29]. In addition, Raman spectroscopy is used to detect exogenous metal ions incorporated into the apatite lattice of bone, which reveals if a specimen's apatite has undergone recrystalisation, raising the possibility that exposed collagen might have also undergone diagenesis (e.g. [24], [30]). This can be investigated because the band energy and width corresponding to the symmetric PO_4_^3-^ vibration in the Raman spectrum is sensitive to changes in the apatite unit cell size due to foreign ion incorporation. [30], [31].

Raman spectroscopy has a fast scan-time and is non-destructive [32]. The technique is also insensitive to the presence of water, and it can be coupled to a light microscope to give sub-micron spatial resolution for dense surface sampling [33]. Raman spectroscopy can be used quantitatively, but to do so requires homogenous calibration standards with known analyte concentrations. When calibration standards are not available, it can be used to provide relative quantification [34]–[36].

The aim of this study is to evaluate how well % collagen yield, % carbon yield and % nitrogen yield as indicators of protein survival, correlate with the Raman spectroscopy evidence for the presence of protein (organic-phosphate ratios). If Raman spectroscopy produced a reasonably reliable “pre-screen” for archaeological bone being considered for stable isotope analysis, it would be a relatively inexpensive, fast and non-destructive test to select bone with the most viable collagen content prior to destructive preparation work and running of mass spectroscopy for dietary stable isotopes from archaeological samples.

## Materials and Methods

### Ban Non Wat

**Figure 1:**
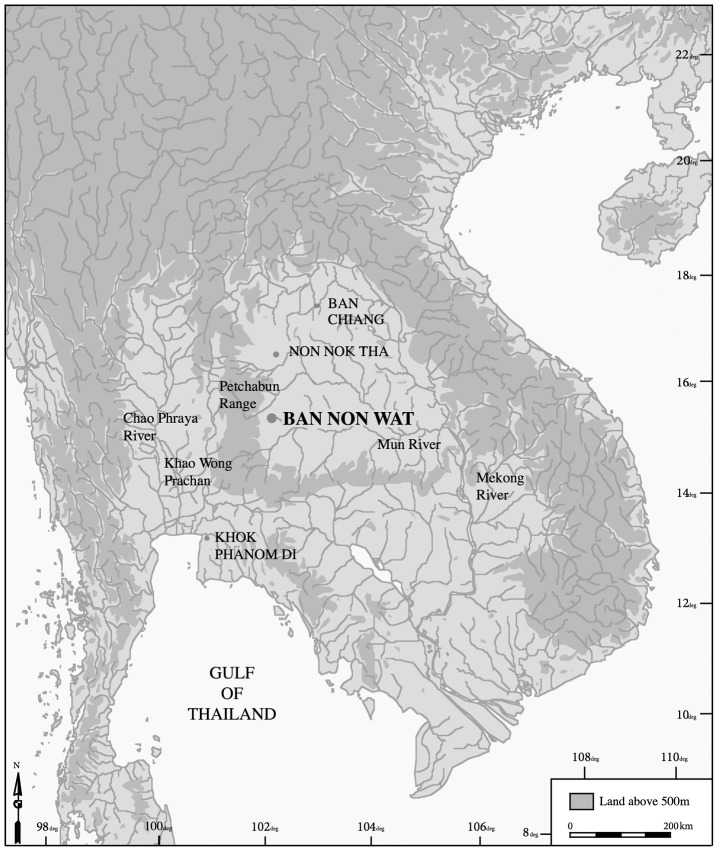
The location of the site of Ban Non Wat in present-day northeast Thailand, and other important archaeological sites (from [38].

The site of Ban Non Wat is situated in the Upper Mun River Valley on the Khorat Plateau, Northeast Thailand (Fig. 1). This site has yielded 630 inhumation burials within an occupation sequence from 3800–1500 BP based on ^14^C AMS dates [37]. The main excavation of this site was carried out under The Origins of Angkor Archaeological Project directed by Professor Charles Higham (University of Otago), Dr Rachanie Thosarat and Dr Amphan Kijngam (Thai Fine Arts Department) over 7 seasons from 2002–2008 [38]. All of the human skeletal remains are stored at the Bone Storage Facility at the 12^th^ Regional Office at Phimai, Northeast Thailand. Full permission from the National Research Council of Thailand (NRCT) and the Thai Fine Arts Department has been granted for this research (NRCT permit number 0002.3/8713). Specimen numbers are designated with burial numbers and Raman IDs, with details given in the results section. During part of a larger study assessing weaning and diet of all subadults excavated from this site (N = 197), bone from 41 of those infants and children were submitted for Raman spectroscopy analysis prior to stable isotope analysis for carbon and nitrogen.

These samples were chosen to represent the different chronological phases of the site, qualified as Neolithic (n = 5), Bronze (n = 23) and Iron Age (n = 13) [38]. Prior to selection for non-destructive Raman and destructive stable isotope analysis all osteological data including age, bone growth, growth disruption and any skeletal and dental pathological information was collected using standard methods outlined in Halcrow [39].

### Raman spectroscopy analysis

For each skeleton, a rib bone was the preferred skeletal element. In cases of poor preservation of ribs, skeletal elements such as segments of long bones were selected. Each bone was scraped with a scalpel to remove any dirt to minimise fluorescence effects, which obscure peaks in the Raman spectrum. The scalpel was soaked briefly in 10% HCl between different bone samples to remove any residues before being rinsed extensively with distilled water and wiped.

Spectra were obtained with a Fourier Transform (FT) Raman spectrometer Equinox 55 Interferometer (Bruker Optics, Ettlingen, Germany) equipped with a FRA-106 Raman accessory and a D418-T liquid nitrogen Ge detector. The 1064 nm excitation laser with a power of 300 mW was used to minimise fluorescence without destroying the organic content of the bone (Nd:YAG laser, Coherent Inc. Santa Clara, USA). A downward-looking objective was used with a spot size of 1 mm to obtain representative spectra. Three replicate spectra (0–3200 cm^−1^) were recorded on different regions of each sample's compact bone. A resolution of 1 cm^−1^ was used to concurrently analyse diagenesis in the mineral phase (results not presented). Each spectrum was the result of the coaddition of 300 scans to produce an acceptable signal-noise ratio. If a strong emission signal was observed before a measurement, that region was not used for data collection. Each Raman spectrum was integrated at 3060–2800 cm^−1^ (C-H symmetric and asymmetric vibrations), 983–930 cm^−1^ (symmetric PO_4_^3−^ vibration) and 566–300 cm^−1^ to obtain band areas.

### Stable Isotope analyses (δ^15^N, δ^13^C, %C, %N)

For the stable isotope determinations, the surfaces of bone samples were mechanically cleaned with a small drill or pared with a scalpel to remove degraded surface material and any adhering soil. The bone was then broken into 2–3 cm fragments and placed in clean plastic 50 ml vials for chemical demineralisation using a modified Longin [40] method with 0.5 M HCl. The bone sample was demineralised for at least 12 hours at room temperature, then each sample was centrifuged, the spent supernatant decanted, and fresh 0.5 M HCl added. This step was repeated until each sample was completely demineralised. The insoluble collagen was isolated by centrifuging and decanting of the supernatant acid, then rinsed to neutral by topping each vial with MilliQ deionised water, centrifuging, and decanting supernatant in repeated washes. The insoluble collagen was gelatinised with 0.01 M HCl at 60°C for 24 h, then double-filtered through Whatman GF/C and 0.45 µm Acrodisc filters. The filtered gelatine was lyophilised to obtain gelatine yields relative to starting weight of physically cleaned bone.

Stable isotope analysis for δ^13^C, δ^15^N, %C and %N was performed at the University of Otago's Isotope Ratio Mass Spectrometry Unit on a Europa Hydra coupled to a Carlo Erba NC 2500, with an average of 0.8 mg of bone gelatine in duplicate samples. All reference materials and internal standards are calibrated and traceable to the international standards V-PDB for ^13^C [41], [42] and AIR for ^15^N [43]. The calibration standard EDTA accompanied each analytical run. Measurements of an EDTA standard within analysis runs (1 EDTA to every six unknowns) indicated variation in analysis of ±0.1‰ at 1σ for δ^15^N and δ^13^C signals.

## Results

### FT-Raman analysis

**Figure 2:**
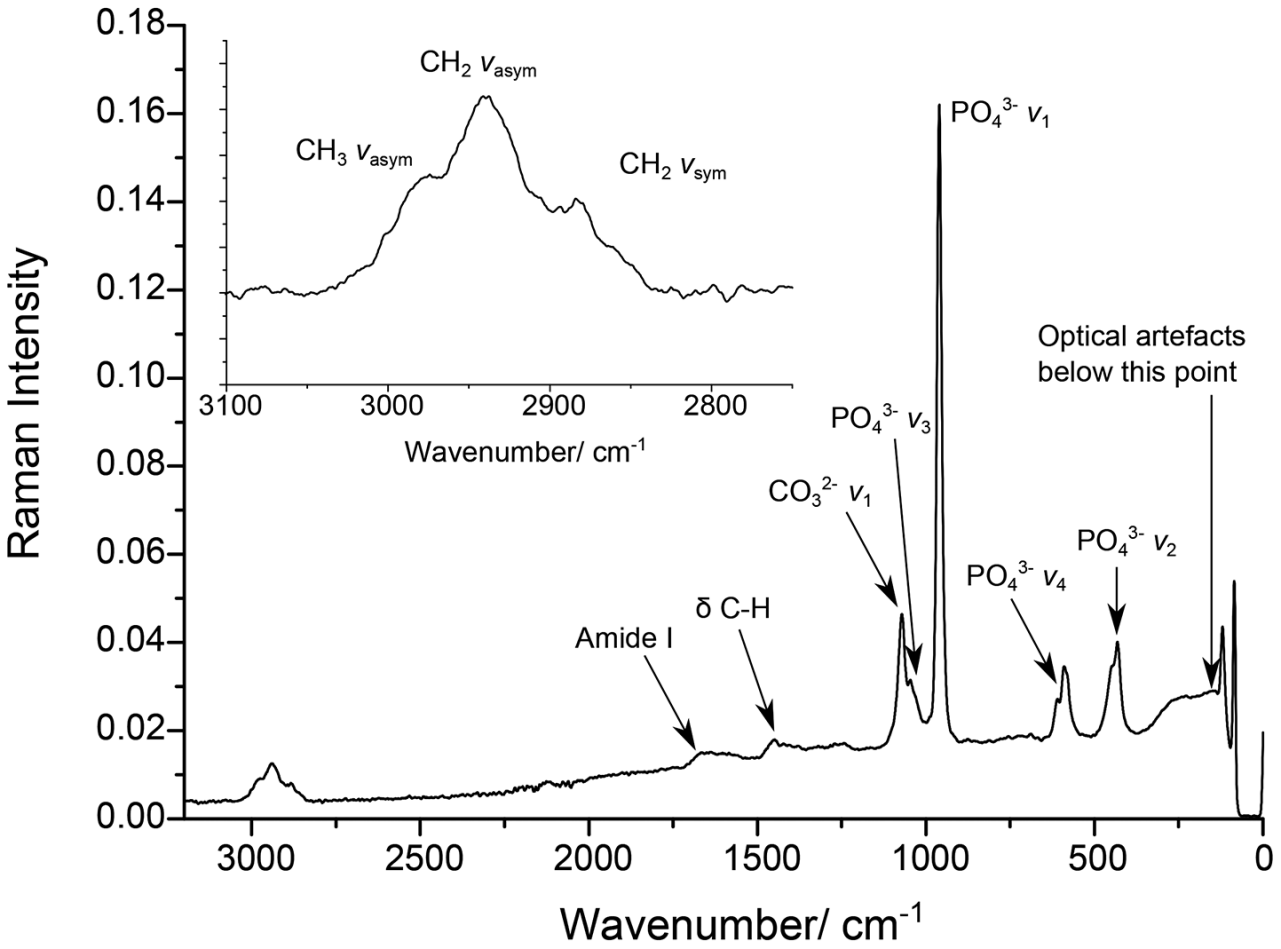
The FT-Raman spectrum of the bone sample from Burial 511 from Ban Non Wat (Raman ID 41), one of the 6 bones that displayed high protein content relative to the other Ban Non Wat specimens. Inset is of the C-H stretch region.

**Figure 3:**
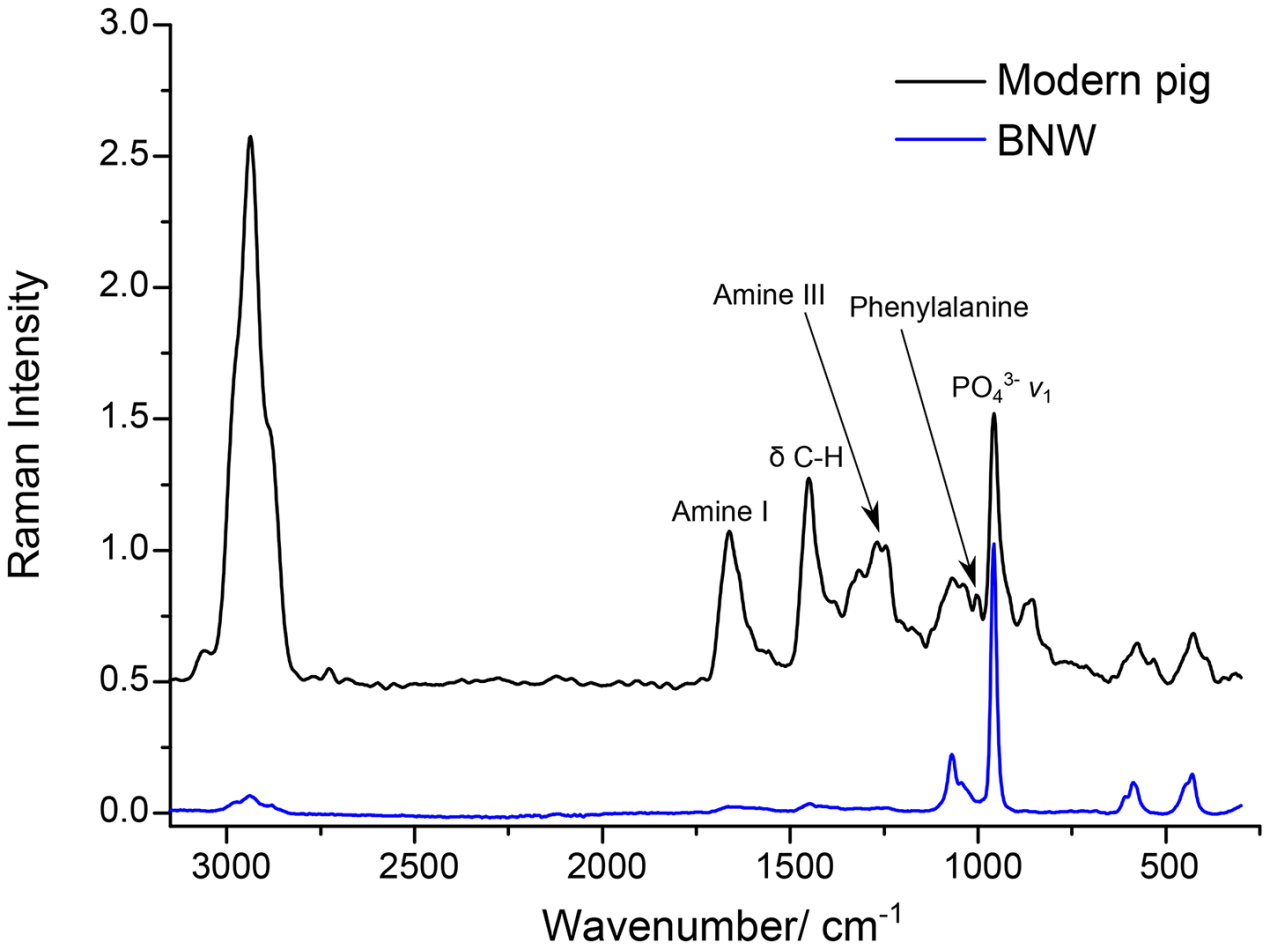
The FT-Raman spectra of a modern pig bone and the bone sample from Burial 511 from Ban Non Wat (Raman ID 41), one of the 6 bones that displayed high protein content relative to the other Ban Non Wat specimens. The spectra have been normalised to the phosphate symmetric stretch and offset for comparison.

All spectra of the bones contained four bands corresponding to the vibrations of the tetrahedral PO_4_^3−^ within the apatite lattice. The vibration at ∼960 cm^−1^ was the most intense and assigned as PO_4_^3−^ stretching symmetrically (*v*_1_) with all four oxygens vibrating in phase (Fig. 2). Carbonate incorporated into the apatite lattice had a symmetric stretch (*v*_1_) present at ∼1071 cm^−1^. Only six bones showed prominent organic vibrational bands, which indicated definite proteinaceous content in the specimens (Raman IDs 22, 24, 28, 29, 33 and 41). The C-H stretch region, composed of symmetric and asymmetric stretching, had three bands in the range of 3060–2800 cm^−1^, which were the primary indicator of organic content. There was also a broad C-H bending vibration (*δ* C-H) at ∼1425 cm^−1^. The remaining samples (n = 12) showed poorly-defined C-H bands with a lesser intensity or no indication of organic content whatsoever (n = 23). Amide I manifested as a broad band at ∼1667 cm^−1^ which is primarily due to the vibrations of carbonyls within the polypeptide and is indicative of the α-helical secondary structure of collagen [28]. Most bones showed this weak, ill-defined amide I feature. In addition to the organic and mineral components of the bone, secondary mineralisation products (calcite and barite) were also observed. This suggests that the apatite portion of the bone within each sample had been exposed to crystallisation processes from ground fluids. The ratio of organic material to apatite was examined using the band areas of the C-H region (3060–2800 cm^−1^) with those of the PO_4_^3−^ (*ν*_1_ and *ν*_2_). The broad band centred at 431 cm^−1^ corresponds to O-P-O bending vibrations (v_2_). Figure 3 shows the Raman spectra of a modern bone, a well-preserved archaeological sheep bone and the Ban Non Wat sample from Figure 2. These spectra demonstrate the poor preservation of the Ban Non Wat samples. In addition to the intense C-H stretching region, the modern bone contains amide III and phenylalanine bands not seen in any Ban Non Wat specimens. The lack of these features is a reflection of the loss of protein in the Ban Non Wat bone.

Previous Raman studies of bone have shown that measurements on two orthogonal planes of orientation affect certain band intensities [27]. The use of a large spot size (diameter 1 mm) was used to mitigate these effects as the sampling area is much larger than domains of orientated collagen fibrils.

### Stable isotope

In Table 1, the % yield of collagen in the BNW bone samples is overall quite low, with a mean of 1.07% (minimum 0.14%; maximum of 6%). Collagen yields of less than 1–2% are usually suspect for adequate stable isotope results. However, due to the nodes of exogenous minerals from burial conditions within the bone structure, starting raw bone weights may not be representative of the bone weight itself, leading to calculation of lower % collagen yield.

**Table 1:** Weight % nitrogen (%N), weight % carbon (%C), % yield of gelatin relative to undemineralised bone starting weight (% collagen), and Raman organic-phosphate ratio [C-H/PO_4_^3−^ (*ν*_1_)] for 31 Ban Non Wat skeletal samples.

Raman ID	Burial no.	Archaeological Phase	% nitrogen w/w	% carbon w/w	C:N	% collagen	Raman ratio
2	573	Neolithic	5.47	21.67	4.6	0.35	0.268
3	254	Neolithic	3.03	16.71	6.4	0.14	0.060
5	563	Neolithic	2.21	6.65	3.5	1.00	0.091
8	538	Bronze	11.37	34.19	3.5	1.19	0.213
9	536	Bronze	6.09	20.34	3.9	0.66	0.311
12	274	Bronze	1.88	7.58	4.7	0.39	0.294
13	130	Bronze	2.86	12.27	5.0	0.28	0.129
14	84	Bronze	5.00	20.51	4.8	0.64	0.204
15	163	Bronze	1.23	10.18	9.7	0.24	0.167
16	425	Bronze	7.26	23.32	3.7	0.30	0.236
17	526	Bronze	3.39	16.78	5.8	0.17	0.154
18	548	Bronze	7.84	25.33	3.8	0.61	0.199
19	559	Bronze	5.87	21.52	4.3	0.26	0.061
20	458	Bronze	10.98	33.50	3.6	0.45	0.240
21	168	Bronze	6.15	22.76	4.3	0.27	0.098
22	141	Bronze	2.94	9.45	3.8	1.30	0.228
23	527	Bronze	6.12	25.34	4.8	0.20	0.154
24	94	Bronze	12.40	35.84	3.4	2.23	0.424
25	280	Bronze	2.72	10.88	4.7	0.31	0.103
26	74	Bronze	0.86	3.82	5.2	0.67	0.087
28	519	Bronze	13.26	37.60	3.3	1.87	0.292
29	139	Bronze	11.25	31.51	3.3	1.49	0.216
30	515	Iron	11.54	34.41	3.5	0.82	0.252
33	584	Iron	14.35	40.77	3.3	2.99	0.597
34	541	Iron	6.29	18.33	3.4	1.47	0.314
35	488	Iron	0.95	3.73	4.6	0.77	0.142
36	484	Iron	7.50	25.89	4.0	0.37	0.244
38	496	Iron	0.51	2.20	5.0	0.60	0.120
40	493	Iron	2.12	6.65	3.7	0.79	0.155
41	511	Iron	12.99	36.50	3.3	3.90	0.393
42	528	Iron	10.56	32.77	3.6	0.79	0.361

The %N, %C, and the atomic CN ratio [Atomic C:N  =  (%C/%N) ×1.1666] from stable isotope analysis are provided to illustrate mass spectrometry indicators of protein survival. The % collagen yield is calculated from bone weight before demineralisation.

Seven burials had nitrogen of 7 µg or less, and produced only carbon delta values and weight percents. An eighth sample, BNW 479, also had a nitrogen value of less than 7 µg did not produce a carbon delta result (Table 2).

**Table 2:** Weight % nitrogen (%N), weight % carbon (%C), % yield of gelatin relative to undemineralised bone starting weight (% collagen), and Raman organic-phosphate ratio [C-H/PO_4_^3−^ (*ν*_1_)] for eight samples for which stable isotope analysis indicates weight % nitrogen at extremely low levels (7 µg or less), including one sample with extremely low weight % carbon.

Raman ID	Burial no.	Archaeological phase	% nitrogen w/w	% carbon w/w	% collagen	Raman ratio
1	560	Neolithic	0.11	2.11	1.88	-
4	80	Neolithic	-	3.43	0.12	0.189
6	146	Bronze	-	6.76	0.27	0.150
7	189	Bronze	2.74	9.28	6.00	-
10	532	Bronze	-	3.44	0.60	0.150
11	550	Bronze	-	1.04	1.75	0.247
31	479	Iron	-	-	0.16	0.208
32	516	Iron	-	3.72	0.40	0.295
37	329	Iron	-	1.85	0.26	0.147
39	481	Iron	-	1.27	0.79	0.145

Two further samples included here (burials 189 and 560) could not have their Raman ratio determined due to fluorescence. The %N, %C, and the atomic CN ratio [Atomic C:N  =  (%C/%N) ×1.1666] from stable isotope analysis are provided to illustrate mass spectrometry indicators of protein survival. The % collagen yield is calculated from bone weight before demineralisation.

### FT-Raman and stable isotope results comparison

**Figure 4:**
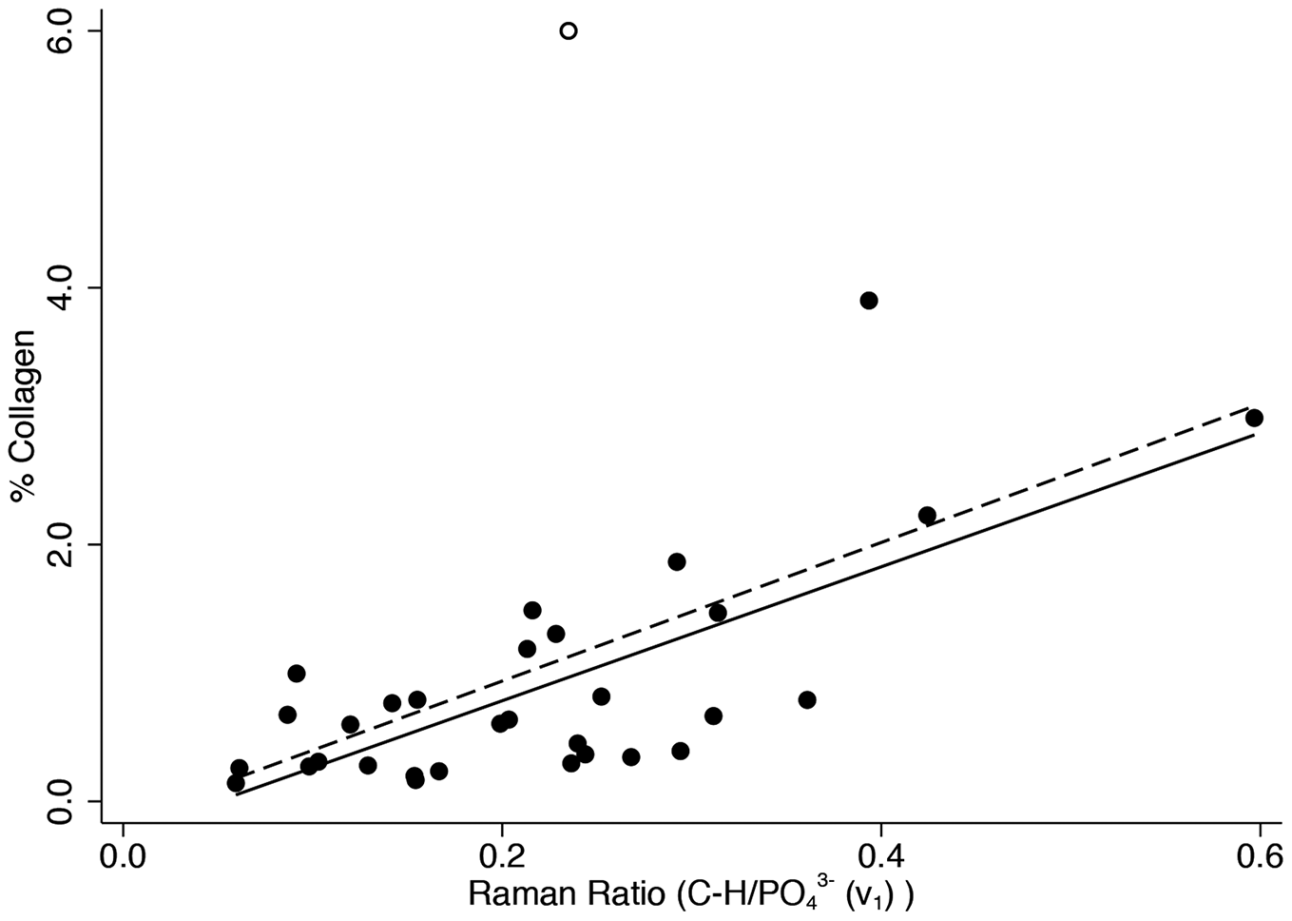
Percentage of collagen content (w/w) versus the organic-phosphate (*ν*_1_) ratio.

**Figure 5:**
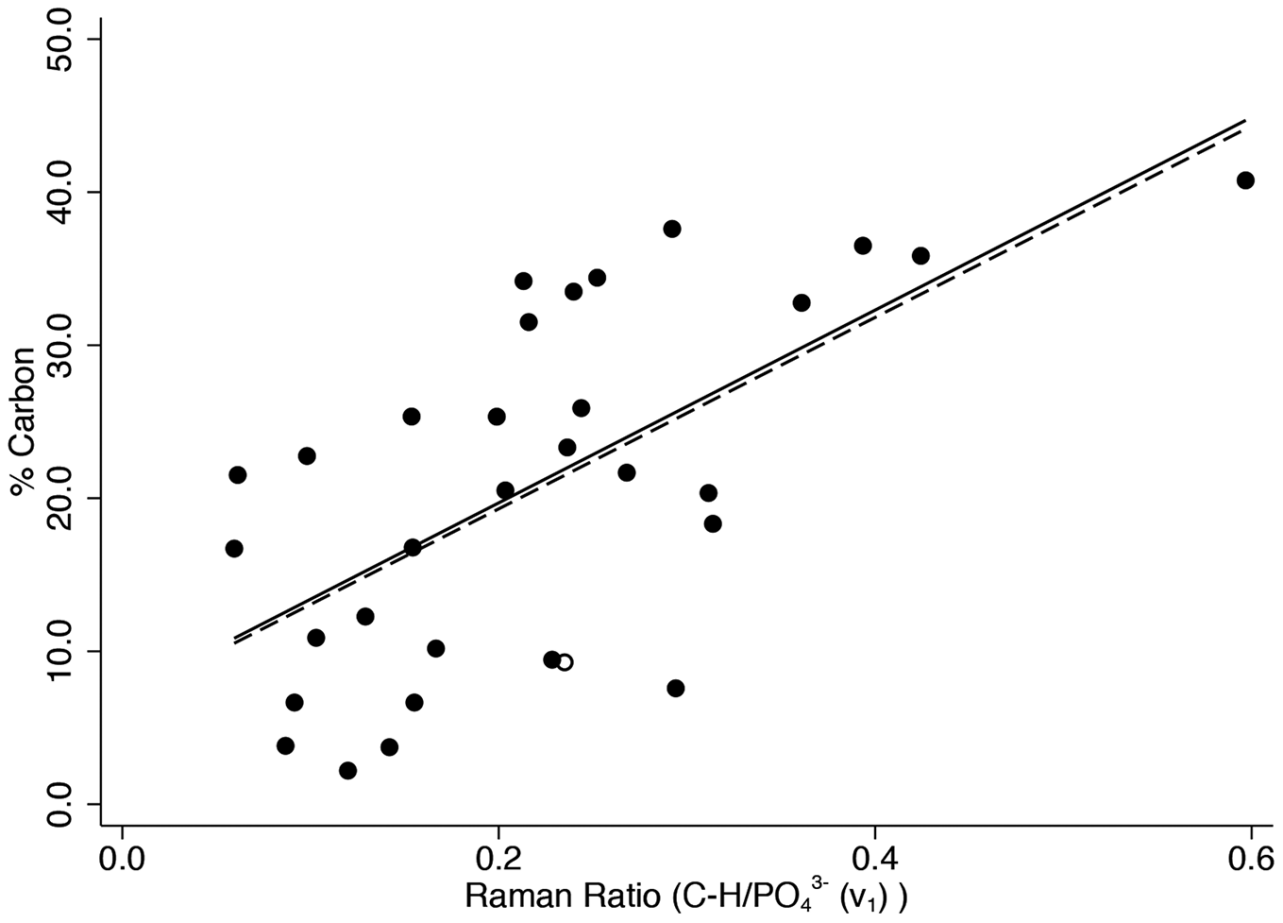
Percentage of carbon content (w/w) versus the organic-phosphate (*ν*_1_) ratio.

**Figure 6:**
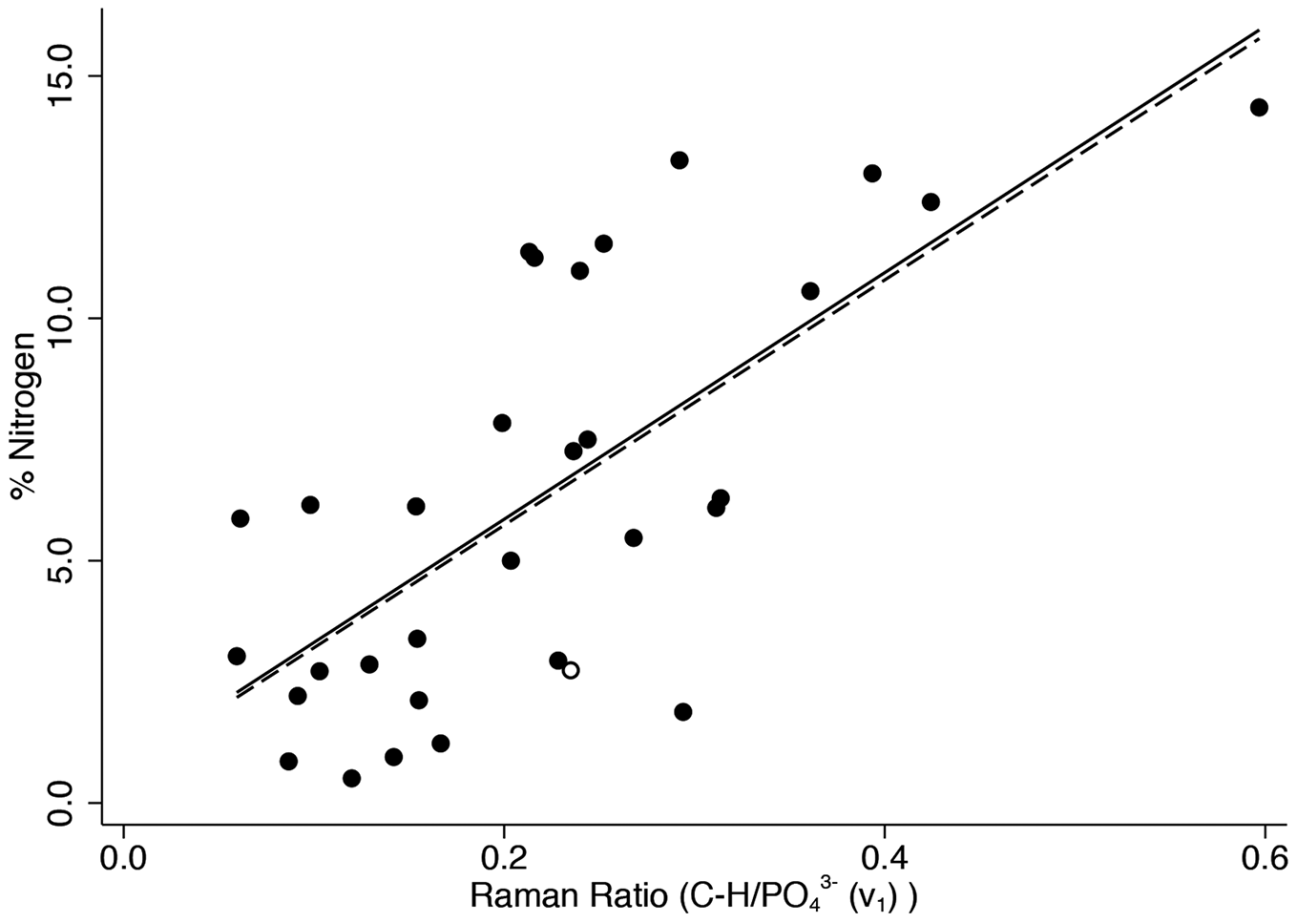
Percentage of nitrogen content (w/w) versus the organic-phosphate (*ν*_1_) ratio.

Figure 4 shows % collagen versus the organic-phosphate ratio (*v*_1_), Figure 5 shows % carbon in the same way, and Figure 6 shows % nitrogen similarly (n = 31 in all graphs). The correlation coefficients are highly statistically significant in all cases (r = 0.716 for collagen, r = 0.630 for carbon, and r = 0.706 for nitrogen, p≤0.001 for all) with approximately or close to half of the variation in each explained by variation in the organic-phosphate ratio (51.2% for collagen, 39.6% for carbon, and 49.8% for nitrogen). Removing the one clear outlier and two leverage points to the right of the graph from the % collagen scatter plot would reduce the coefficient of determination to 17.8% (p = 0.025) and removing the one leverage point to the right of the carbon graph would only slightly reduce R^2^ to 36.1%. Removing the one leverage point to the right of the graph from the % nitrogen scatter plot would reduce R^2^ to 43.8% (p<0.001). There was no evidence before or after removing these points that the association between the organic-phosphate ratio and % carbon was stronger than with % collagen (difference in correlation coefficients p = 0.555 before and p = 0.379 after removing the unusual points), or for % nitrogen than with % collagen (difference in correlation coefficients p = 0.942 before and p = 0.214 after removing the unusual points), or for % carbon than with % nitrogen (difference in correlation coefficients p = 0.606 before and p = 0.712 after removing the unusual points).

## Discussion

In this experiment FT-Raman spectroscopy organic-phosphate ratio results are compared with % collagen yield from the whole bone sample, and % carbon and % nitrogen from isotopic analysis. Because statistically significant correlations between these variables were found, it is feasible that Raman spectroscopy could be useful in the selection of bone for subsequent destructive chemical treatment in the course of stable isotope analysis. There was no evidence (whether or not the odd data points are kept or removed) that there is a stronger association between the organic-phosphate ratio with % collagen, % carbon or % nitrogen. All samples with greater than 1% collagen yields showed C-H stretches from visual examination of the Raman spectra (n = 12). Considering bones that have % collagen yields below 1–2% are undesirable for isotope analysis, visual inspection of the spectra allowed viable bones to be identified. The Raman spectra allowed qualitative presence or absence of protein to be determined which can save time, expense and preserve sample integrity before other techniques are applied. The relationship between the Raman spectroscopy qualitative organic scores and % collagen and nitrogen preservation found in this study could be further validated using archaeological bone samples with differing preservation.

It has been shown by previous studies (e.g. [44]) that collagen content is not lost uniformly, and exogenous ions are not incorporated uniformly through the bone. Some of the variability in the correlation between the Raman collagen and actual collagen yield from isotopic preparation may be attributable to subsampling. This is because Raman scattering primarily occurs on the surface of samples with penetration depth of a few hundred micrometres for a 1064 nm laser [45], [46]. In contrast, the weight percent of carbon and nitrogen by mass spectrometry was determined from collagen samples derived from the whole bone, which may have variable preservation. However, homogeneity on the surface of the bone as indicated in the triplicate spectra appeared reasonable with the exception of three bones. In addition, as noted in the methods, in order to record a representative composition, each spectrum gathered data from a spot 1 mm in diameter. Further research should be conducted into the extent of variable collagen preservation within bone specimens especially those which are poorly preserved.

These results are consistent with previous research, which showed that there is a relationship between bone recrystalisation and collagen preservation [47]. King's [25] study of diagenesis at Ban Non Wat identified secondary mineralisation products of calcite and barite in their Raman analysis of adult bones from BNW, which was also supported with tentative identification of calcite and barite crystals using SEM. The present study's results showed that secondary mineralisation products (calcite and barite) were also present, suggesting that the apatite portion of the bone within each sample had been exposed to recrystallisation processes from ground water.
